# Application of a Speedy Modified Entropy Method in Assessing the Complexity of Baroreflex Sensitivity for Age-Controlled Healthy and Diabetic Subjects

**DOI:** 10.3390/e21090894

**Published:** 2019-09-14

**Authors:** Ming-Xia Xiao, Chang-Hua Lu, Na Ta, Wei-Wei Jiang, Xiao-Jing Tang, Hsien-Tsai Wu

**Affiliations:** 1 School of Computer Science and Information Engineering, Hefei University of Technology, No.193 Tunxi Road, Hefei, Anhui 230009, China; xiao_mx@nmu.edu.cn (M.-X.X.); jsdzlch@hfut.edu.cn (C.-H.L.); jiangww@hfut.edu.cn (W.-W.J.); 2 School of Electrical and Information Engineering, North Minzu University, No. 204 North Wenchang Street, Yinchuan, Ningxia 750021, China; ta_na@nmu.edu.cn; 3 School of Science, Ningxia Medical University, No. 1160 Shengli Street, Yinchuan, Ningxia 750004, China; tangxj@nxmu.edu.cn; 4 Department of Electrical Engineering, Dong Hwa University, No. 1, Sec. 2, Da Hsueh Rd., Shoufeng, Hualien 97401, Taiwan, China

**Keywords:** autonomic nervous function, heart rate variability (HRV), baroreflex sensitivity (BRS), photo-plethysmo-graphy (PPG), digital volume pulse (DVP), percussion entropy index (PEI)

## Abstract

The percussion entropy index (PEI_orginal_) was recently introduced to assess the complexity of baroreflex sensitivity. This study aimed to investigate the ability of a speedy modified PEI (i.e., PEI_NEW_) application to distinguish among age-controlled subjects with or without diabetes. This was carried out using simultaneous photo-plethysmo-graphy (PPG) pulse amplitude series and the R wave-to-R wave interval (RRI) series acquired from healthy subjects (Group 1, number = 42), subjects diagnosed as having diabetes mellitus type 2 with satisfactory blood sugar control (Group 2, number = 38), and type 2 diabetic patients with poor blood sugar control (Group 3, number = 35). Results from PEI_orginal_ and multiscale cross-approximate entropy (MCAE) were also addressed with the same datasets for comparison. The results show that optimal prolongation between the amplitude series and RRI series could be delayed by one to three heartbeat cycles for Group 2, and one to four heartbeat cycles for Group 3 patients. Group 1 subjects only had prolongation for one heartbeat cycle. This study not only demonstrates the sensitivity of PEI_NEW_ and PEI_orginal_ in differentiating between Groups 2 and 3 compared with MCAE, highlighting the feasibility of using percussion entropy applications in autonomic nervous function assessments, it also shows that PEI_NEW_ can considerably reduce the computational time required for such processes.

## 1. Introduction

A depressed autonomic nervous function may lead to cardiovascular system damage, resulting in the occurrence and development of various cardiovascular diseases [1]. A frequency domain analysis of heart rate variability (HRV) using electrocardiography (ECG) has been used over the past 20 years to assess autonomic function [2]. The low-frequency-to-high-frequency power ratio (LHR) is considered to reflect the balance between sympathetic and parasympathetic activities [3,4]. 

In the past decade, the autonomic nervous system has been shown to play a key role in the physiological regulation of blood pressure and the heartbeat interval. Qualitatively, baroreflex refers to a physiological phenomenon in which a decrease in blood pressure shortens the RR interval (RRI), and an increase in blood pressure prolongs the RRI. Baroreflex sensitivity (BRS) refers quantitatively to the degree of matching between changes in the RRI and blood pressure during a cardiac cycle [5,6]. Quantitatively, two identical increases (or decreases) in blood pressure during two successive cardiac cycles are unlikely to produce two identical prolongations (or reductions) in RRI. In individuals with a blunted baroreflex, two successive increases in blood pressure may not even produce two successive RRI prolongations. The dynamic interactions of blood pressure and heartbeat interval contain very important information about autonomic nervous function. Thus, as a nonlinear interaction approach to evaluate autonomic nervous system activities, BRS can be reflexed by autonomic nervous function [7,8,9].

However, the synchronized physiological signal acquisition for blood pressure and heartbeat interval is not practical for real-time applications [10,11]. Luckily, the amplitude time series acquired noninvasively through digital volume pulse (DVP) signals from photo-plethysmo-graphy (PPG) has been found to correlate well with changes in blood pressure [12,13,14]. PPG pulse amplitudes are more easily acquired than blood pressure signals. A previous study [15] using synchronized PPG pulse amplitude series and the RRI series highlighted the application of multiscale cross-approximate entropy (MCAE) in noninvasively identifying changes in autonomic nervous function in persons with or without diabetes. The results of autonomic nervous function assessments from LHR, the pulse–pulse-interval-and-amplitude ratio (PAR), and multiscale entropy (MSE) were also computed for comparison in [15].

In addition, among the three one-dimensional approaches to autonomic nervous function assessment (i.e., LHR, the Poincaré index (SD1/SD2 ratio, SSR) and the small-scale multiscale entropy index (MEI_SS_)), only the MEI_SS_ has been shown to successfully discriminate among nondiabetic subjects, as well as those with diabetes with or without satisfactory blood sugar control [16]. In contrast to MCAE, the percussion entropy index (PEI) [16] is based upon a simple method of assessing the similarity in the fluctuation patterns of two synchronized time series (i.e., PPG pulse amplitude series and ECG RR interval signals) to evaluate the BRS regulation capacity of a physiological system of the human body for autonomic nervous function assessment. For example, in [16], the possibility of using PEI to assess autonomic sensitivity by counting the percussion numbers between the two fluctuating time series of DVP and RRI with shift numbers of 1–5 was assessed [17].

The BRS delay between RRI and blood pressure series in the computation of the BRS parameters under various blood pressure perturbation techniques was discussed in [18,19,20], considering not only cardiac BRS, but also sympathetic BRS. However, most of the above studies and corresponding references focused upon healthy young humans or upper middle-aged subjects, not on diabetes patients. Previous studies [21,22,23] have demonstrated that there may be different effects for different shift numbers among nondiabetic subjects and diabetics with or without satisfactory blood sugar control, because the BRS regulation capabilities between these groups are quite different. On the other hand, using time and frequency domain methods, previous studies [24,25] have demonstrated that young subjects with type 1 diabetes mellitus experience decreased sympathetic and parasympathetic activities (i.e., BRS reduction), and a lower compliance between blood pressure and heart rate fluctuations compared with healthy young subjects. In 2011, Professor Javorka et al. [26] reported that in addition to the increase in time delay within BRS regulation in young patients with type 1 diabetes mellitus, the level of similarity between blood pressure and heart rate fluctuations was significantly reduced. Therefore, we conjecture that the percussion rate of the amplitude series and RR interval signals would reach expectations in a shorter time (i.e., with a small shift number) for healthy humans than for those with diabetes. In other words, a new modified percussion entropy index (i.e., PEI_NEW_) with a smaller shift number in the percussion rate computation for healthy humans compared to those with diabetes, could be found [21,22,23,24,25,26]. 

The objective of the current study was to test two hypotheses: (1) That the prolongation between the amplitude series and RRI series could be more seriously delayed for type 2 diabetics and elderly patients with poor blood sugar control, and (2) that this new approach (PEI_NEW_) would significantly reduce the computation time compared with the past PEI method. In other words, the aim of the present study was to validate the hypothesis that nondiabetic elderly subjects or type 2 diabetic elderly subjects with satisfactory blood sugar control could have lower PEI computation time for shorter shift numbers.

The rest of the paper is organized as follows: Section 2 describes the study population; experimental procedure; study protocol; details on data acquisition, including the RRI sequence and fingertip PPG amplitude sequence (i.e., RRI and Amp) and processes of percussion entropy indices (i.e., PEI_original_ and PEI_NEW_); and the computation times for the comparison and statistical analysis. In Section 3, the choice of the optimal shift number for PEI computation is justified, followed by a comparison of the three relative parameters for autonomic function assessment. Section 4 and Section 5 respectively contain the discussion and conclusions related to the findings, as well as suggestions for future work.

## 2. Materials and Methods

### 2.1. Study Population and Experimental Procedure

#### 2.1.1. Study Population and Grouping

Seventy-eight type 2 diabetic patients were recruited from the diabetic outpatient clinic of Hualien Hospital (Hualien City, Taiwan) from July 2009 to March 2012. They were all diagnosed by either a glycosylated hemoglobin (HbA1c) concentration greater than 6.5% or a fasting glucose concentration higher than 126 mg/dL [27]. They had also received regular treatment in the clinic for more than two years. Of the 78 patients, five were excluded due to unstable waveform data acquisition. In addition, 42 age-controlled healthy subjects were recruited from a health examination program during the same period and from the same hospitals. The remaining 115 volunteers were then divided into three groups: Healthy subjects (Group 1, age range: 41–78 years, number = 42), 38 subjects diagnosed as having diabetes mellitus type 2 with satisfactory blood sugar control (Group 2, age range: 41–82 years, 6.5% ≦ HbA1c < 8%), and 35 type 2 diabetic patients with poor blood sugar control (Group 3, age range: 44–77 years, HbA1c ≧ 8% [28]) (Table 1). The study was approved by the Institutional Review Board (IRB) of Hualien Hospital and Ningxia Medical University (Yinchuan City, Ningxia Province, PRC)—Hospitals. All subjects gave written informed consent.

#### 2.1.2. Experimental Procedure

In this study, all subjects rested in a supine position in a quiet, temperature-controlled room at 25 ± 1 °C for 4 min prior to the 30 min measurements. Before the measurements were taken, a questionnaire was given to each subject to obtain detailed information on their general health condition and medical history. Age, gender and demographic data, including body height, body weight and waist circumference, were also recorded. Blood samples were obtained from all subjects after 8 h of fasting to determine the levels of serum triglyceride, high-density lipoproteins, fasting blood glucose and HbA1c. Systolic and diastolic blood pressure were measured over the left arm of the supine subjects with an automated oscillometric device (BP3AG1, Microlife, Taipei, Taiwan). Subsequently, a self-developed, six-channel electrocardiography-pulse wave velocity (ECG-PWV)-based system, which was previously described, was used to acquire 1000 successive recordings of photo-plethysmo-graphy (PPG) and ECG waveforms within 30 min [29]. Briefly, the six-channel ECG-PWV system consists of synchronized PPG and ECG measurements. Digital volume pulses of PPG were acquired by an infrared sensor and attached to the left index finger. The PPG signals were amplified with an INA128 (Texas Instruments, Dallas, TX, USA), and then transmitted to a second-order band-pass filter and another low-pass filter. The pulse signals were then transmitted to a second-order band-pass filter at frequencies of 0.48–10 Hz and a low-pass filter at frequencies below 10 Hz. Subsequently, the ECG signals were acquired in lead II and transmitted to a notch filter set at 59–61 Hz and a band-pass filter at frequencies of 0.98–19.4 Hz. In order to store and analyze the sampled waveforms of the PPG and ECG signals, a USB-6009 DAQ (National Instruments, Austin, TX, USA) converted these two signals to digital signals and transmitted them to a personal computer with a sampling frequency of 500 Hz. After this, we used the LabVIEW 8.6 package (National Instruments, Austin, TX, USA) for data saving and analysis. 

### 2.2. Study Protocol

ECG and PPG signals were simultaneously acquired from all subjects. Two previous parameters, percussion entropy index (PEI_orginal_) and multiscale cross-approximate entropy (MCAE), with average values from scales 1 to 10, were then calculated from the Amp and RRI time series for each subject. A speedy modified percussion entropy index (PEI_NEW_) was developed for autonomic function assessment after choosing the optimal delay prolongation between the above two time series. The associations of the computational parameters (i.e., MCAE, PEI_original_ and PEI_NEW_) with the demographic (i.e., age), anthropometric (i.e., body height, body weight, waist circumference and body mass index), hemodynamic (i.e., systolic and diastolic blood pressures), and serum biochemical (i.e., fasting blood glucose and glycated hemoglobin, high- and low-density lipoprotein cholesterol, triglycerides and total cholesterol) parameters of the three groups of subjects were then calculated and analyzed. 

### 2.3. A Speedy Modified Entropy Method for Assessing the Complexity of Baroreflex Sensitivity

#### 2.3.1. Percussion Entropy Index, PEI_original_

Time series of the DVP waveform amplitude (Amp = {Amp(1), Amp(2), …, Amp(1001)}) and RRI (RRI = {RRI(1), RRI(2), …, RRI(1006)}) were simultaneously captured from 1,006 successive and stable cardiac cycles with PPG and ECG, respectively, for each subject:

Amp = {Amp(1), Amp(2), Amp(3), …, Amp(1001)},(1)

RRI = {RRI(1), RRI(2), RRI(3), …, RRI(1006)}.(2)

(1) Taking BRS regulation into account, the binary sequence transformations for Amp and RRI were computed:
  (3) $$B_{\mathrm{Amp}}=\{a_{1}\;a_{2}\;a_{3}\;a_{000}\},$$
  (4) $$\text{where},\;{\text{a}}_{\text{i}}=\{\begin{array}{c} 0,\;\;\mathrm{Amp}(\text{i}+1)\leq \mathrm{Amp}(\text{i})\\ 1,\;\;\mathrm{Amp}(\text{i}+1)>\mathrm{Amp}(\text{i}) \end{array}$$
  (5) $$B_{\mathrm{RRI}}=\{r_{1}\;r_{2}\;r_{3}\;r_{1005}\},$$
  (6) $$\text{where},\;{\text{r}}_{\text{i}}=\{\begin{array}{c} 0,\;\;\mathrm{RRI}(\text{i}+1)\leq \mathrm{RRI}(\text{i})\\ 1,\;\;\mathrm{RRI}(\text{i}+1)>\mathrm{RRI}(\text{i}) \end{array}$$
(2) The n − m + 1 vectors of patterns for BAmp and BRRI, each of size m, were defined, and these were composed as follows:
  B_Amp_(i) = {a_i_, a_i+1_,…, a_i+m-1_}, 1 ≤ i ≤ n − m + 1 (7)
  For s = 1–5 (i.e., shift numbers), the series B_RRI_,
  B_RRI_(i+s) = {r_i+s_, r_i+s +1_,…, r_i+s+m-1_}, 1 ≤ i ≤ n − m + 1, s = 1 to 5. (8)
(3) The percussion rate (i.e., the similarity in the pattern of fluctuation) for BAmp(i) and BRRI(i+s) was counted with the given m.
  Then, the total match number of B_Amp_(i) and B_RRI_(i+s) was counted with the same pattern (i.e., the percussion number) and divided by the total number of vectors of patterns (n – m – s + 1) to obtain the percussion rate, which was expressed as
  (9) $${\;P}_{s}^{m}=\frac{1}{\left(n-m-s+1\right)}\overset{n-m-s+1}{\underset{i=1}{\sum }}\mathrm{count}\left(i\right).$$
(4) The logarithm of the sum of percussion rates (Pms) from shift numbers 1–5 (i.e., s = 1, 2, 3, 4, 5) gave
  (10) $$\varphi^{m}\left(n\right)=ln\left(\sum_{s=1}^{5}P_{s}^{m}\right),\;ln:\mathrm{natural}\;\mathrm{logarithmic}\;\mathrm{operation}.$$
(5) The embedded dimension was increased to (m + 1), and (9) and (10) changed to
  (11) $$P_{s}^{m+1}=\frac{1}{\left(n-m-s+2\right)}\overset{n-m-s+2}{\underset{i=1}{\sum }}\mathrm{count}\left(i\right),$$
  (12) $$\varphi^{m+1}\left(n\right)=ln\left(\sum_{s=1}^{5}P_{s}^{m+1}\right).$$
(6) According to a previous study [16], the percussion entropy index was defined as
  (13) $$\mathrm{PEI}\;\mathrm{original}\;\left(m,\;n\right)=\varphi^{m}\left(n\right)-\varphi^{m+1}\left(n\right),$$
  (14) $$=ln\left[\frac{\sum_{s=1}^{5}P_{s}^{m}}{\sum_{s=1}^{5}P_{s}^{m+1}}\right].$$

**Figure 1:**
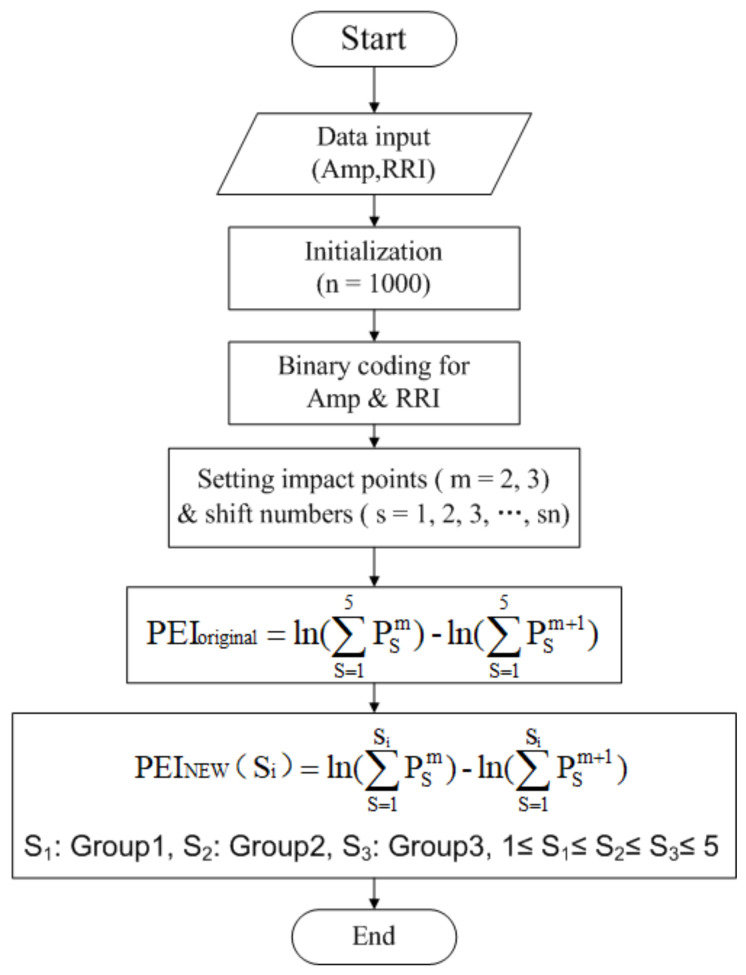
Flow chart of two percussion entropy index computations. Two synchronized photo-plethysmo-graphy (PPG) pulse amplitude series (Amp) and RR interval (RRI) series were acquired. The computational length of the data was 1000. Taking baroreflex sensitivity (BRS) regulation into account, binary sequence transformations were carried out for Amp and RRI. After the impact point and three optimal shift numbers had been set, the percussion entropy index (PEI_original_) and the new PEI (PEI_NEW_) were computed.

As in [16], where the possibility of using PEI_original_ to assess autonomic function by counting the percussion numbers between the two fluctuating time series of Amp and RRI with a fixed shift number of 1 to 5 for every group, the parameters in this study were set at m = 2 and n = 1000 (Figure 1). 

In the next section, we describe the derivation of a new modified percussion entropy index (i.e., PEI_NEW_) with a smaller shift number in percussion rate computation for healthy humans compared with diabetics [24,25,26,30,31].

#### 2.3.2. A Speedy Modified Percussion Entropy Index, PEI_NEW_

● Signal processing and calculation of PEI_NEW_

We hypothesized that the BRS delay between the amplitude series and RRI series could be more seriously delayed for patients with diabetes and poor blood sugar control. Therefore, PEI_original_ in (14) was modified to

(15) $${\mathrm{PEI}}_{\mathrm{NEW}}\left(m,n,\mathrm{Si}\right)=ln\left[\frac{\;\sum_{s=1}^{\mathrm{Si}}P_{s}^{m}}{\sum_{s=1}^{\mathrm{Si}}P_{s}^{m+1}}\right].$$

In addition, the parameters in this study were also set to m = 2 and n = 1000 for comparison (Figure 1). Thus, (15) was changed to (16) to make it easy to understand:

(16) $${\mathrm{PEI}}_{\mathrm{NEW}}\left(\mathrm{Si}\right)=ln\left[\frac{\sum_{s=1}^{\mathrm{Si}}P_{s}^{2}}{\sum_{s=1}^{\mathrm{Si}}P_{s}^{3}}\right].$$

Based on the findings in [24,25,26,30,31], the BRS regulation capability differs among groups. The optimal prolongation in (16) between amplitude series and RRI series could be delayed for patients with diabetes (i.e., Group 2) and poor blood sugar control (i.e., Group 3). Hence, we assumed the following: the optimal shift number was expressed as S_1_ for Group 1, S_2_ for Group 2, and S_3_ for Group 3, where

1 ≦ S_1_ ≦ S_2_ ≦ S_3_ ≦5. (17)

● Criteria for selecting the optimal shift number

The Pearson correlation and Bland–Altman plot were then adopted to determine the optimal values of S_1_, S_2_, and S_3_ in (17). 

A. For S_1_ selection for Group 1, the following process is required:

1. Assuming S_1_ = 1, calculate the Pearson correlation coefficients (r) of PEI_NEW_ (1) and PEI_NEW_ (2); PEI_NEW_ (1) and PEI_NEW_ (3); PEI_NEW_ (1) and PEI_NEW_ (4); and PEI_NEW_ (1) and PEI_NEW_ (5).
2. If {r > 0.8, and is statistically significant, (*p* < 0.05)} and {PEI_NEW_ (1) and PEI_NEW_ (2) show good agreement}, then stop (S_1_ = 1). Subsequently, go to S_2_ selection; otherwise, go to the next step.
3. Assuming S_1_ = 2, calculate the Pearson correlation coefficients (r) of PEI_NEW_ (2) and PEI_NEW_ (3); PEI_NEW_ (2) and PEI_NEW_ (4); and PEI_NEW_ (2) and PEI_NEW_ (5).
4. If {r > 0.8 and is statistically significant, (*p* < 0.05)} and {PEI_NEW_ (2) and PEI_NEW_ (3) show good agreement}, then stop (S_1_ = 2). Subsequently, go to S_2_ selection; otherwise, go to the next step.
5. Assuming S_1_ = 3, calculate the Pearson correlation coefficients (r) of PEI_NEW_ (3) and PEI_NEW_ (4) and PEI_NEW_ (3) and PEI_NEW_ (5).
6. If {r > 0.8 and is statistically significant, (*p* < 0.05)} and {PEI_NEW_ (3) and PEI_NEW_ (4) show good agreement}, then stop (S_1_ = 3). Subsequently, go to S_2_ selection; otherwise go to the next step.
7. Assuming S_1_ = 4, calculate the Pearson correlation coefficient (r) of PEI_NEW_ (4) and PEI_NEW_ (5).
8. If {r > 0.8 and is statistically significant, (*p* < 0.05)} and {PEI_NEW_ (4) and PEI_NEW_ (5) show good agreement}, then stop (S_1_ = 4). Subsequently, go to S_2_ selection; otherwise, stop.

B. For S_2_ selection for Group 2, start from S_2_ = 1 and follow the steps for S_1_ selection; 

C. For S_3_ selection for Group 3: start from S_3_ = 1 and follow the steps for S_1_ selection.

### 2.4. Computation Times for Comparison 

The computation times of MCAE, PEI_original_, and PEI_NEW_ for all test subjects were obtained and compared. For this purpose, a workstation was used with the following specifications: ASUSPRO Notebook with Intel (R) Core (TM) i5-4210U CPU@1.70 GHz 2.40 GHz, Windows 10 Home. In terms of signal analysis software, the computation package MATLAB 2016a (MathWorks Inc., Natick, Massachusetts, USA) was adopted. Two functional instructions, “tic” and “toc”, from MATLAB were utilized to determine the CPU computation times. 

### 2.5. Statistical Analysis

All values in the tables are denoted as the mean ± SD. The Statistical Package for the Social Sciences (SPSS, version 14.0 for Windows, SPSS Inc. Chicago, IL, USA) was utilized for all statistical analyses. The one-sample Kolmogorov–Smirnov test was adopted to test the normality of the distribution, and then the homoscedasticity of the variables was verified. 

To identify significant prolongations between amplitude series and RRI series for the three groups, the study adopted the Pearson correlation test with Bonferroni correction to determine the optimal shift number of each group, and then a Bland–Altman plot was utilized for further verification of the agreement and assessment of statistical significance. The significance of differences in anthropometric, hemodynamic and determined parameters (i.e., MCAE, PEI_original_, and PEI_NEW_) among different groups were determined using independent sample *t*-tests with Bonferroni correction. The correlations between risk factors and compared parameters for different groups were computed using the Pearson correlation test. A *p*-value < 0.017 was regarded as statistically significant.

## 3. Results

Results from the two old indices, PEI_orginal_ and MCAE, were first computed using the same datasets for comparison. Subsequently, the optimal BRS delay between amplitude series and the RRI series was identified for each group. Finally, the performance and high-speed characteristics of PEI_NEW_ were verified.

### 3.1. Optimal Prolongation between the Amplitude Series and RRI Series for the Three Groups 

#### 3.1.1. A Simple Way to Estimate the Delay between Amp and RRI

**Figure 2:**
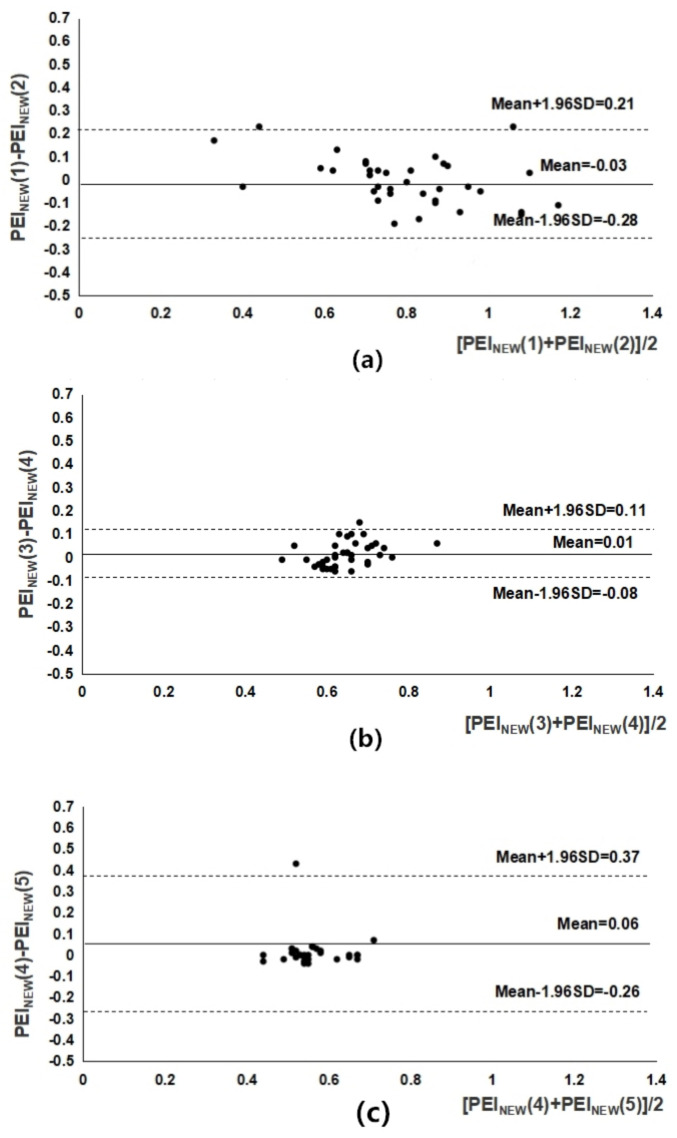
Bland–Altman plots showing good agreement between two PEI_NEW_ sequences in (16) for (**a**) case **A**, (**b**) case **H**, and (**c**) case **J**. The mean difference and the limits of agreement are also indicated.

1. S_1_ Selection for Group 1. As shown in Table 2, two PEI_NEW_ sequences in (16) were computed from cases **A**–**D** for Group 1, followed by the Pearson correlation calculation for the two sequences. For example, we obtained two time series, PEI_NEW_(1) and PEI_NEW_(2), in case **A** for Group 1 subjects, which were very highly correlated (r = 0.91) and statistically significant (p = 0.01). Then, the optimal shift number for Group 1 was expressed as 1 (i.e., S_1_ = 1).
2. S_2_ Selection for Group 2. For Group 2 subjects, as in Step 1, we obtained two time series, PEI_NEW_(3) and PEI_NEW_(4), in case **H** for Group 2 subjects, which were very highly correlated (*r* = 0.84 > 0.8) and statistically significant (*p* < 0.00) (Table 2). After checking the Bland–Altman plot (Figure 2b), the optimal shift number for Group 2 was expressed as 3 (i.e., S_2_ = 3).
3. S3 Selection for Group 3. For Group 3 subjects, as in Step 1, we obtained two time series, PEI_NEW_(4) and PEI_NEW_(5), in case **J** for Group 3 subjects, which were very highly correlated (*r* = 0.87 > 0.8) and statistically significant (*p* < 0.00) (Table 2). After checking the Bland–Altman plot (Figure 2c), the optimal shift number for Group 3 was expressed as 4 (i.e., S3 = 4).

#### 3.1.2. Reproducibility Analysis for PEI_NEW_ and PEI_orginal_ for All Subjects

We tested the reproducibility [28] of the PPG and RRI signals by calculating the coefficients of variation for PEI_NEW_ and PEI_orginal_, which were 2.74% and 14.90%, respectively.

#### 3.1.3. Correlation between PEI_NEW_ and PEI_orginal_ for the Three Groups

**Figure 3:**
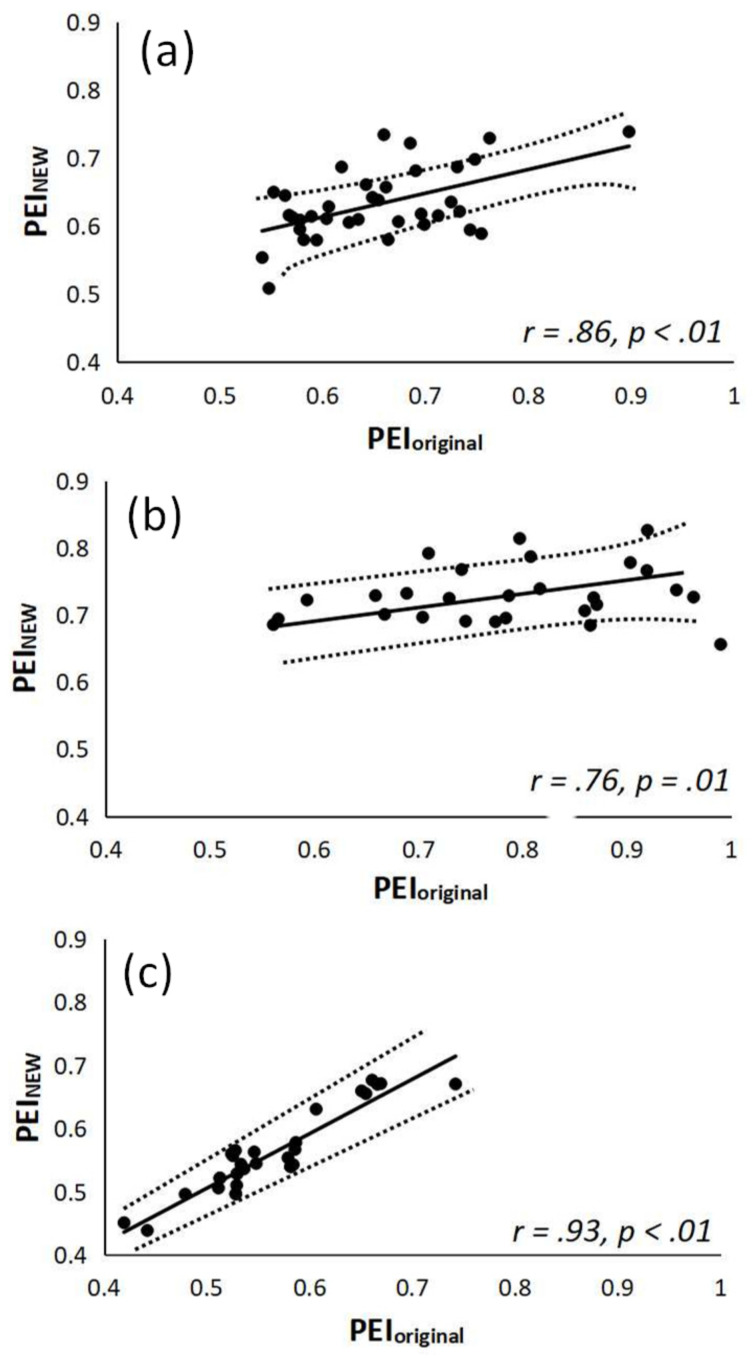
(**a**) Positive correlation between PEI_NEW_ (i.e., S_1_ = 1) and PEI_orginal_ for Group 1 subjects (*r* = 0.86, *p* < 0.00); (**b**) positive correlation between PEI_NEW_ (i.e., S_2_ = 3) and PEI_orginal_ for Group 2 subjects (*r* = 0.76, *p* = 0.01); (**c**) positive correlation between PEI_NEW_ (i.e., S_3_ = 4) and PEI_orginal_ for Group 3 subjects (*r* = 0.93, *p* < 0.00). Group 1: Healthy subjects; Group 2: Diabetic subjects with good blood sugar control; Group 3: Diabetic subjects with poor blood sugar control. The regression line depicts the 95% confidence interval.

Figure 3 shows the regression of PEI_NEW_ and PEI_orginal_ for the three groups with a 95% confidence interval and the correlation coefficient (*r*). Figure 3 was added to verify the hypothesis S_1_ ≦ S_2_ ≦ S_3_. The correlation study tested three groups of subjects. The values of PEI_NEW_ (i.e., S_1_ = 1 in (16)) were significantly correlated with PEI_orginal_ (i.e., shift numbers 1–5 in (14)) for Group 1 subjects (*r* = 0.86, *p*<0.00, Figure 3a). The values of PEI_NEW_ (i.e., S_2_ = 3 in (16)) were significantly correlated with PEI_orginal_ (i.e., S = 1–5 in (14)) for Group 2 patients (*r* = 0.76, *p* = 0.01, Figure 3b). As shown in Figure 3c, the values of PEI_NEW_ (i.e., S_3_ = 4 in (16)) were significantly highly correlated with PEI_orginal_ (i.e., S = 1–5 in (14)) for Group 3 patients (r = 0.93, *p* < 0.00).

### 3.2. Comparison among MCAE, PEI_original_, and PEI_NEW_ for Autonomic Function Assessment in All Testing Subjects 

The results of comparing the two previous computational parameters (i.e., MCAE and PEI_original_) with PEI_NEW_ for autonomic function assessment among the three groups of subjects are shown in Table 3. Although the value of MCAE was significantly higher in Group 1 compared with Group 2 subjects (p < 0.017), there was no notable difference between Groups 2 and 3. On the other hand, PEI_original_, and especially PEI_NEW_, showed highly significant differences among the three groups (p < 0.001) (Table 3).

### 3.3. Correlations of Demographic, Anthropometric, Hemodynamic, and Serum Biochemical Data with MCAE, PEI_original_, and PEI_NEW_

Table 4 illustrates the correlations between parameters associated with metabolic syndrome, including demographic, anthropometric, hemodynamic and serum biochemical data, with MCAE, PEI_original_ and PEI_NEW_. Significant associations were noted between MCAE and the serum triglyceride concentration, as well as between MCAE and fasting blood sugar (both p < 0.017). Significant associations were noted between PEI_original_ and waist circumference, serum triglyceride concentration, glycated hemoglobin and fasting blood sugar, as well as between PEI_NEW_ and waist circumference, serum triglyceride concentration, glycated hemoglobin and fasting blood sugar in all subjects, regardless of diabetic status (Table 4).

### 3.4. Computation Time for MCAE, PEI_original_, and PEI_NEW_ in All Testing Subjects 

Computation times for MCAE, PEI_original_ and PEI_NEW_ from all the subjects were computed and compared (Table 5). Significantly shorter computation times were noted for PEI_NEW_ compared with those for MCAE and PEI_original_ for each group (Table 5). The computation times for PEI_original_ could not be distinguished among the three groups, while the computation times of PEI_NEW_ for Group 1 were all highly significantly reduced compared with those for the other two groups (Table 5 and Table 6). 

## 4. Discussion

In recent decades, several studies [2,3,4] have used frequency domain parameters for noninvasive autonomic nervous function assessment in clinical patients. Considering that baroreflex sensitivity is an indicator of autonomic function [5,6,7,8,9], as well as previous findings showing a good correlation between real-time changes in blood pressure and DVP signals amplitudes [12,13,14], this study investigated the possibility of assessing autonomic sensitivity by quantifying the increase or decrease fluctuation matches between the two time series of DVP and RRI with shift numbers of 1 to s_n_ (e.g., s_n_ ≦ 5). This hypothesis was based on the findings of previous reports, which showed a delay of BRS of between one to five heartbeats [16,17,26].

Previously, in [16], the impact of diabetes and blood sugar control on autonomic nervous function was assessed by comparing the percussion rate of two synchronized physiological time series to fluctuations (i.e., synchronized PPG pulse amplitude series and RRI series) in subjects with or without diabetes. In contrast to one-dimensional frequency (i.e., LHR) and time (i.e., SSR) domain analyses of HRV, the percussion entropy index (i.e., PEI_original_) was able to discriminate among subjects with and without diabetes, as well as those with or without satisfactory blood sugar control. Second, PEI_original_ was shown to be the only index with significant correlations between acute and chronic blood sugar control parameters. The results highlight the conspicuous sensitivity of this index in detecting diabetes-associated autonomic dysfunction. However, a fixed BRS delay of the RRI (i.e., 1–5) was used for PEI computation in all age-controlled subjects. Despite its creative applicability, the computation load of PEI_original_ could be large for real-time applications (Table 5).

It is well known that diabetes is associated with blunted baroreflex regulation and suppressed autonomic activity [17,32]. The evaluation of baroreflex sensitivity is a nonlinear approach to the assessment of autonomic nervous activity [33]. The complexity of baroreflex regulations in healthy and diabetic subjects is considered a ubiquitous phenomenon in physiology that allows subjects to adapt to external perturbations by preserving homeostasis. This originates from specific features of the system, such as its nonlinearity, through physiological networks [34]. Previous studies [18,19,20] demonstrated the time delay between the RRI and blood pressure series in the computation of the BRS under various blood pressure perturbation techniques. The most relevant fluctuations in the heart rate period occur at around six seconds or faster [30]. It has also been shown that the baroreflex values change more dramatically in young healthy subjects than in elderly hypertensive subjects and the increased efficiency of the baroreflex control at night might explain the nocturnal BP reduction.

These results are consistent with the known loss of high-frequency modulation of the baroreflex with age and disease (i.e., hypertension) [31]. Unfortunately, most of the above studies and corresponding references did not focus on the optimal delay between RRI and blood pressure values for the diabetes. 

The BRS regulation capability of different groups (e.g., subjects without diabetes as well as those with or without satisfactory blood sugar control) is quite different [21,22,23]. Young type 1 diabetics showed decreases in parasympathetic and sympathetic activities (i.e., BRS reduced), and an overall variability of the autonomic nervous system in [24]. In [25], young type 1 diabetics were shown to have autonomic nervous system behavior that tends to be random (i.e., with low compliance between blood pressure and heart rate fluctuations), compared with healthy young subjects using different time and frequency domain methods. Another previous study [26] demonstrated that young type 1 diabetics had a larger BRS delay and similarity between blood pressure and heart rate fluctuations. Thus, the aim of this study was to determine the optimal BRS delay between RRI and blood pressure values (“Amp series” in this study) for different subjects (e.g., diabetic and elderly individuals). The optimal BRS delay between the amplitude and RRI series could be delayed one to three heartbeat cycles for diabetic subjects with well-controlled blood sugar (i.e., 1–3) and by one to four heartbeat cycles for those with poor blood sugar control (i.e., 1–4). Group 1 subjects, who were age-matched non-diabetics, had an optimal BRS delay of one heartbeat cycle (Table 2 and Figure 2). For indirect verification of the hypothesis (i.e., S_1_ ≦ S_2_ ≦ S_3_), the current study not only showed that the values of PEI_NEW_ significantly correlated with PEI_original_ (Figure 3), but also demonstrated the good reproducibility for PEI_NEW_. Accordingly, the computation times for PEI_NEW_ were all highly significantly reduced for Group 1 compared with those for the other two groups (Group 1 vs. Group 2 vs. Group 3: 3.80 ± 0.29 vs. 7.87 ± 0.33 vs. 8.11 ± 0.39 ms) (Table 5). In conclusion, this study demonstrated that elderly type 2 diabetics and patients with poor blood sugar control have a larger BRS delay and complex fluctuations between the PPG amplitude series and RRI (Table 2, Figure 2 and Figure 3). Moreover, although diabetic neuropathy was found to be a more important determining factor of spontaneous baroreflex sensitivity assessment than carotid elasticity in type 2 diabetics in [35], blood sugar control was not considered. It is worth mentioning that PEI_original_, and especially PEI_NEW_, were successfully differentiated among the three groups with highly significant differences in our study (p < 0.001) (Table 3). In addition, the difference between MCAE and PEIs (i.e., PEI_original_ and PEI_NEW_) is that the former assesses the degree of probability of two parameters within the same defined region after data detrending, normalization and continuous shifting [15,36], whereas the latter is a simple way to evaluate the similarity in the fluctuation patterns (i.e., increase or decrease) of two synchronized PPG pulse amplitude series and RRI series to assess the adaptive capacity of a living system [16]. This could be another reason for the CPU time reduction (Table 6). 

The current study has its limitations. Firstly, the number of subjects recruited was relatively small. Nevertheless, highly significant associations between percussion entropy indices and CPU time parameters were still significant. Secondly, we only focused on three parameters (i.e., MCAE, PEI_original_, and PEI_NEW_) using synchronized PPG pulse amplitude series and RRI series, and direct assessment of BRS with either invasive or noninvasive means was not adopted for comparison with the results of the present study. Finally, the values of MCAE, PEI_original_, and PEI_NEW_ could be used as features in a group classification task by using simple machine learning algorithms (such as random forest and logistic classifiers) in the future.

## 5. Conclusions

This study represents the first attempt to investigate the satisfactory application of a speedy modified entropy parameter (i.e., PEI_NEW_) for the assessment of baroreflex sensitivity complexity in healthy elderly and diabetic subjects related to type 2 diabetes-associated autonomic function changes. Our findings suggest that both PEI_NEW_ and PEI_original_ could serve as novel, noninvasive biomarkers for discriminating diabetes-related changes in BRS regulation, which is of importance for preventive care. Taking into account the shorter percussion computation time, PEI_NEW_ demonstrated the feasibility and enhanced sensitivity of autonomic nervous function applications in real-time data analysis, characteristics which are of vital importance for the development of noninvasive instruments to compute the complexity of synchronized physiological signals in the human body.

## Figures and Tables

**Table 1:** Summary of anthropometric, demographic, hemodynamic, and serum biochemical information of the study subjects.

Parameters	Group 1 Number: 42 Female/Male (24/18)	Group 2 Number: 38 Female/Male (17/21)	Group 3 Number: 35 Female/Male (12/23)
Age, years	56.73 ± 3.80	60.05 ± 8.29	58.08 ± 11.33
Body height, cm	163.50 ± 8.33	163.59 ± 7.98	162.41 ± 5.18
Body weight, kg	65.00 ± 13.80	71.60 ± 11.82	79.60 ± 16.22
WC, cm	81.75 ± 11.80	94.35 ± 9.75 ^**^	101.01 ± 13.49 ^††^
BMI, kg/m^2^	24.16 ± 4.07	26.53 ± 2.82 ^*^	29.81 ± 6.15
SBP, mmHg	116.46 ± 15.59	125.66 ± 18.02	125.69 ± 10.19
DBP, mmHg	73.69 ± 9.73	75.06 ± 12.36	76.35 ± 4.26
PP, mmHg	42.40 ± 10.70	51.55 ± 11.88	50.30 ± 12.08
HDL, mg/dL LDL, mg/dL	53.21 ± 20.80 122.35 ± 29.50	44.04 ± 9.89 94.36 ± 21.90	40.50 ± 9.62 118.10 ± 28.91
Cholesterol, mg/dL Triglyceride, mg/dL	192.45 ± 40.00 98.06 ± 85.36	170.81 ± 31.05 112.92 ± 39.92	199.10 ± 34.62 185.89 ± 74.90
HbA1c, %	5.69 ± 0.37	6.93 ± 0.39 ^**^	9.25 ± 1.60 ^††^
FBS, mg/dL	93.99 ± 10.65	127.45 ± 25.70 ^**^	176.91 ± 68.51 ^††^

Group 1: Healthy subjects; Group 2: Diabetic subjects with good blood sugar control; Group 3: Diabetic subjects with poor blood sugar control. All values are presented as mean ± SD. WC: Waist circumference; BMI: Body mass index; SBP: Systolic blood pressure; DBP: Diastolic blood pressure; PP: Pulse pressure; HDL: High-density lipoprotein cholesterol; LDL: Low-density lipoprotein cholesterol; HbA1c: Glycosylated hemoglobin; FBS: Fasting blood sugar. ^*^p < 0.017 Group 1 vs. Group 2, ^**^ *p* < 0.001 Group 1 vs. Group 2, **^††^** *p* < 0.001 Group 2 vs. Group 3. A *p*-value < 0.017 was classified as statistically significant.

**Table 2:** Univariate analysis of the correlation of two PEI_NEW_ sequences in (16) for subjects from Groups 1–3.

Case	Group 1		Group 2		Group 3	
	*r*	*p*	*r*	*p*	*r*	*p*
**A**	**0.91**	**0.01**	0.13	0.45	0.47	0.01
**B**	0.10	0.55	0.06	0.73	0.22	0.25
**C**	−0.36	0.02	0.21	0.23	0.05	0.81
**D**	−0.14	0.39	−0.03	0.88	0.13	0.47
**E**	-	-	0.78	0.00	0.76	0.00
**F**	-	-	0.36	0.03	0.34	0.06
**G**	-	-	0.45	0.01	0.37	0.04
**H**	-	-	**0.84**	**0.00**	0.50	0.01
**I**	-	-	-	-	0.41	0.02
**J**	-	-	-	-	**0.87**	**0.00**

Group 1: Healthy subjects; Group 2: Diabetic subjects with satisfactory blood sugar control; Group 3: Diabetic subjects with poor blood sugar control. **A**: PEI_NEW_(1) and PEI_NEW_ (2); **B**: PEI_NEW_(1) and PEI_NEW_(3); **C**: PEI_NEW_(1) and PEI_NEW_(4); **D**: PEI_NEW_(1) and PEI_NEW_(5); **E**: PEI_NEW_(2) and PEI_NEW_(3); **F**: PEI_NEW_(2) and PEI_NEW_(4); **G**: PEI_NEW_(2) and PEI_NEW_(5); **H**: PEI_NEW_(3) and PEI_NEW_(4); **I**: PEI_NEW_(3) and PEI_NEW_(5); **J**: PEI_NEW_(4) and PEI_NEW_(5); 0 ≦ |r| ≦ 0.3: Correlation of low significance; 0.3 ≦ |r| ≦ 0.7: Correlation of moderate significance; 0.7 ≦ |r| ≦ 1: Highly significant correlation. The significance of these correlations was determined with the Pearson correlation.

**Table 3:** Comparison of computational parameters for autonomic function assessment in three groups of testing subjects.

Parameters	Group 1 (N = 42)	Group 2 (N = 38)	Group 3 (N = 35)
**MCAE**	0.83 ± 0.08	0.74 ± 0.09 *	0.75 ± 0.05
**PEI_original_**	0.73 ± 0.04	0.63 ± 0.07 **	0.56 ± 0.09 ^†^
**PEI_NEW_**	0.82 ± 0.04	0.65 ± 0.01 **	0.58 ± 0.01 ^††^

Group 1: healthy subjects; Group 2: diabetic subjects with satisfactory blood sugar control; Group 3: diabetic subjects with poor blood sugar control. Values are expressed as mean ± SD. MCAE: Multiscale Cross-Approximate Entropy; PEI_original_: percussion entropy index in (14); PEI_NEW_: speedy percussion entropy index in (16). * *p* < 0.017: Group 1 vs. Group 2; ** *p* < 0.001: Group 1 vs. Group 2; **^†^** *p* < 0.017 Group 2 vs. Group 3; **^††^** *p* < 0.001 Group 2 vs. Group 3.

**Table 4:** Associations of demographic, anthropometric, hemodynamic and serum biochemical data with computational parameters for autonomic function assessment in all subjects.

	PEI_NEW_		PEI_original_		MCAE	
	*r*	*p*	*r*	*p*	*r*	*p*
**Age (years)**	0.32	0.24	0.07	0.49	0.08	0.46
**BH (cm)**	0.01	0.90	0.16	0.09	0.19	0.08
**BW (kg)**	–0.18	0.06	–0.33	0.02	0.18	0.11
**WC** **(cm)**	–0.25	**0.01**	–0.42	**0.00**	0.00	0.98
**BMI (kg/m^2^)**	–0.20	**0.04**	–0.25	**0.01**	0.08	0.49
**SBP (mmHg)**	–0.04	0.67	–0.01	0.89	0.16	0.16
**DBP (mmHg)**	–0.03	0.76	–0.04	0.69	0.19	0.91
**PP (mmHg)**	–0.04	0.72	0.01	0.90	0.09	0.45
**HDL (mg/dL)**	0.09	0.35	0.13	0.20	0.02	0.84
**LDL (mg/dL)**	–0.15	0.14	–0.20	0.04	-0.16	0.18
**Cholesterol (mg/dL)**	0.10	0.33	–0.09	0.37	0.17	0.08
**Triglyceride (mg/dL)**	–0.27	**0.01**	–0.31	**0.00**	–0.21	**0.00**
**HbA1c (%)**	–0.45	**0.00**	–0.57	**0.00**	–0.16	0.18
**FBS (mg/dL)**	–0.29	**0.00**	–0.53	**0.00**	–0.73	**0.00**

BH: Body height; BW: Body weight; WC: Waist circumference; BMI: Body mass index, SBP: Systolic blood pressure; DBP: Diastolic blood pressure; PP: Pulse pressure; HDL: High-density lipoprotein cholesterol; LDL: Low-density lipoprotein cholesterol; HbA1c: Glycated hemoglobin; FBS: Fasting blood sugar; MCAE: Multiscale cross-approximate entropy; PEI_original_: Percussion entropy index in (14); PEI_NEW_: Speedy percussion entropy index in (16). |*r*| ≦ 0.3: Correlation of low significance; 0.3 ≦ |*r*| ≦ 0.7: Correlation of moderate significance; 0.7 ≦ |*r*| ≦ 1: Highly significant correlation. The significance of these correlations was determined with the Pearson correlation.

**Table 5:** Comparison of CPU times for MCAE, PEI_original_ and PEI_NEW_ for all testing subjects.

	Group 1 (N = 42)	Group 2 (N = 38)	Group 3 (N = 35)
**CPU time for MCAE (ms)**	23.61 ± 0.87	20.93 ± 0.63 *	21.62 ± 0.77
**CPU time for PEI_original_ (ms)**	14.17 ± 0.53	13.95 ± 0.78	13.65 ± 0.66
**CPU time for PEI_NEW_ (ms)**	3.80 ± 0.29	7.87 ± 0.33 **	8.11 ± 0.39

Group 1: healthy subjects; Group 2: diabetic subjects with satisfactory blood sugar control; Group 3: diabetic subjects with poor blood sugar control. Values are expressed as mean ± SD. * *p* < 0.017: Group 1 vs. Group 2; ** *p* < 0.001: Group 1 vs. Group 2. MCAE: Multiscale Cross-Approximate Entropy; PEI_original_: percussion entropy index in (14); PEI_NEW_: speedy percussion entropy index in (16).

**Table 6:** Comparison of CPU times for MCAE, PEI_original_ and PEI_NEW_ under different subject combinations.

	MCAE	PEI_original_	PEI_NEW_
**Group 1**	23.61 ± 0.87	14.17 ± 0.53 **	3.80 ± 0.29 ^††^
**Group 2**	20.93 ± 0.63	13.95 ± 0.78 *	7.87 ± 0.33 ^†^
**Group 3**	21.62 ± 0.77	13.65 ± 0.66 *	8.11 ± 0.39 ^†^
**Group 2&Group 3**	21.31 ± 0.69	13.81 ± 0.75 *	7.95 ± 0.38 ^†^
**Group 1&Group 2&Group 3**	22.03 ± 0.81	14.00 ± 0.65 **	5.88 ± 0.34 ^††^

MCAE: Multiscale Cross-Approximate Entropy; PEI_original_: percussion entropy index in (14); PEI_NEW_: speedy percussion entropy index in (16). Group 1: healthy subjects; Group 2: diabetic subjects with satisfactory blood sugar control; Group 3: diabetic subjects with poor blood sugar control. Values are expressed as mean ± SD (ms). * *p* < 0.017: MCAE vs. PEI_original_; ** *p* < 0.001: MCAE vs. PEI_original_. **^†^** *p* < 0.017: PEI_original_ vs. PEI_NEW_; **^††^** *p* < 0.001: PEI_original_ vs. PEI_NEW_.
